# MD2 blockade prevents modified LDL-induced retinal injury in diabetes by suppressing NADPH oxidase-4 interaction with Toll-like receptor-4

**DOI:** 10.1038/s12276-021-00607-w

**Published:** 2021-04-19

**Authors:** Huaicheng Chen, Tao Yan, Zongming Song, Shilong Ying, Beibei Wu, Xin Ju, Xi Yang, Jia Qu, Wencan Wu, Zongduan Zhang, Yi Wang

**Affiliations:** 1 Chemical Biology Research Center, School of Pharmaceutical Sciences, Wenzhou Medical University, Wenzhou, Zhejiang China; 2 Eye Hospital and School of Ophthalmology and Optometry, Wenzhou Medical University, Wenzhou, 325027 Zhejiang China; 3 Department of Ophthalmology, The Fourth Affiliated Hospital, Zhejiang University School of Medicine, Yiwu, Zhejiang China; 4 Henan Eye Institute, Henan Eye Hospital, Henan Provincial People’s Hospital, People’s Hospital of Zhengzhou University, Wenzhou, China

**Keywords:** Obesity, Type 2 diabetes

## Abstract

Modified LDL-induced inflammation and oxidative stress are involved in the pathogenesis of diabetic retinopathy. Recent studies have also shown that modified LDL activates Toll-like receptor 4 (TLR4) to mediate retinal injury. However, the mechanism by which modified LDL activates TLR4 and the potential role of the TLR4 coreceptor myeloid differentiation protein 2 (MD2) are not known. In this study, we inhibited MD2 with the chalcone derivatives L2H17 and L6H21 and showed that MD2 blockade protected retinal Müller cells against highly oxidized glycated-LDL (HOG-LDL)-induced oxidative stress, inflammation, and apoptosis. MD2 inhibition reduced oxidative stress by suppressing NADPH oxidase-4 (NOX4). Importantly, HOG-LDL activated TLR4 and increased the interaction between NOX4 and TLR4. MD2 was required for the activation of these pathways, as inhibiting MD2 prevented the association of NOX4 with TLR4 and reduced NOX4-mediated reactive oxygen species production and TLR4-mediated inflammatory factor production. Furthermore, treatment of diabetic mice with L2H17 significantly reduced LDL extravasation in the retina and prevented retinal dysfunction and apoptosis by suppressing the TLR4/MD2 pathway. Our findings provide evidence that MD2 plays a critical role in mediating modified LDL-induced cell injury in the retina and suggest that targeting MD2 may be a potential therapeutic strategy.

## Diabetes: Specific protein inhibitors could reduce blindness

Blocking the activity of a protein involved in triggering inflammation and oxidative stress in the retina may reduce diabetes-induced visual impairment and blindness. Besides hyperglycemia, plasma lipids and lipoproteins have also been proposed as potential risk factors for diabetic retinopathy. However, the precise mechanisms controlling low-density lipoprotein-induced retinal damage are unclear. In experiments on mice, Yi Wang at Wenzhou Medical University in Wenzhou, China, and co-workers found that retinal injury caused by highly oxidized glycated-LDL is mediated by the myeloid differentiation protein 2 (MD2). Blocking MD2 with an inhibitor suppressed two key signaling pathways, reducing the production of reactive oxygen species and inflammatory signaling proteins in the retina. Further investigations are needed, but the team believe MD2 could be a vital therapeutic target for diabetic retinopathy.

## Introduction

Diabetic retinopathy is the leading cause of visual impairment and blindness in the working population^1^. Hyperglycemia-induced inflammation^2,3^ and oxidative stress^4^ contribute greatly to the progression of diabetic retinopathy^5,6^. In addition, persistent hyperglycemia causes early functional alterations including reduced blood flow, neuronal dysfunction in response to light stimulation, and blood–retinal barrier (BRB) leakage^7–9^. Plasma proteins, including low-density lipoproteins (LDL), may cross the BRB and leak into the retinal parenchyma from damaged vessels^9^. This process may induce various inflammatory responses, as LDL can be modified by oxidation and glycation in the retina^9^. Although there is supporting evidence, the detailed underlying mechanisms of oxidative stress and inflammation induced by modified LDL are not yet fully understood.

Oxidized LDL (ox-LDL) is known to activate inflammatory responses in various cell types, including retinal Müller cells^10^. Müller cells are the principal glia of the retina and are found almost throughout the entire retina. Müller cells form processes around retinal vessels, modulate neuronal activity and glucose metabolism, control blood flow and maintain the BRB^11^. Hyperglycemia-induced production of interleukin-1β (IL-β), interleukin-6 (IL-6), tumor necrosis factor-α (TNF-α), and chemokine ligand-2 (CCL2) by Müller cells is linked to retinal cell death^12^. Müller cells also secrete growth factors that cause vascular alterations in diabetic retinopathy^13^. Therefore, targeting Müller cells may offer a therapeutic benefit in diabetic retinopathy.

Ox-LDL itself or through the oxidation of lipids and proteins in the retina may generate damage-associated molecular patterns (DAMPs). DAMPs can activate the pattern-recognition receptors (PRRs) present on Müller cells^14^. Toll-like receptor-4 (TLR4) is perhaps the most studied PRR and is expressed in Müller cells^15^. TLR4 polymorphisms are associated with an inflammatory state in diabetes^16,17^. Recent studies have implicated the TLR4 signaling pathway in diabetes-induced inflammation in the retina^18–20^. Activation of TLR4, at least by lipopolysaccharide, requires a coreceptor protein known as myeloid differentiation protein-2 (MD2). Our laboratory has previously shown that MD2 also binds to palmitic acid in obesity models and angiotensin II in hypertension models to mediate inflammatory injury^21,22^. Whether MD2 plays a role in modified LDL-induced inflammatory responses in retinal cells is not currently known.

In the present study, we examined the role of MD2 in modified LDL-induced inflammatory responses in Müller cells using two small-molecule inhibitors of MD2: L2H17^23^ and L6H21^24^. We previously showed that these chalcone derivative MD2 inhibitors attenuated obesity-induced heart^25^ and renal dysfunction^26^. The results from the present study showed that MD2 blockade prevented highly oxidized glycated-LDL (HOG-LDL)-induced TLR4 activation in Müller cells. Inhibition of the TLR4 signaling pathway reduced apoptosis, oxidative and endoplasmic reticulum stress, and the induction of proinflammatory cytokines in Müller cells. Using a type 2 diabetic mouse model, we further showed that MD2 inhibition normalized diabetes-induced retinal dysfunction, LDL extravasation, oxidative stress, and inflammation. These findings provide new mechanistic insights into the role of modified LDL in diabetic retinopathy and the potential therapeutic effect of MD2 inhibition.

## Materials and methods

### General reagents

**Fig. 1:**
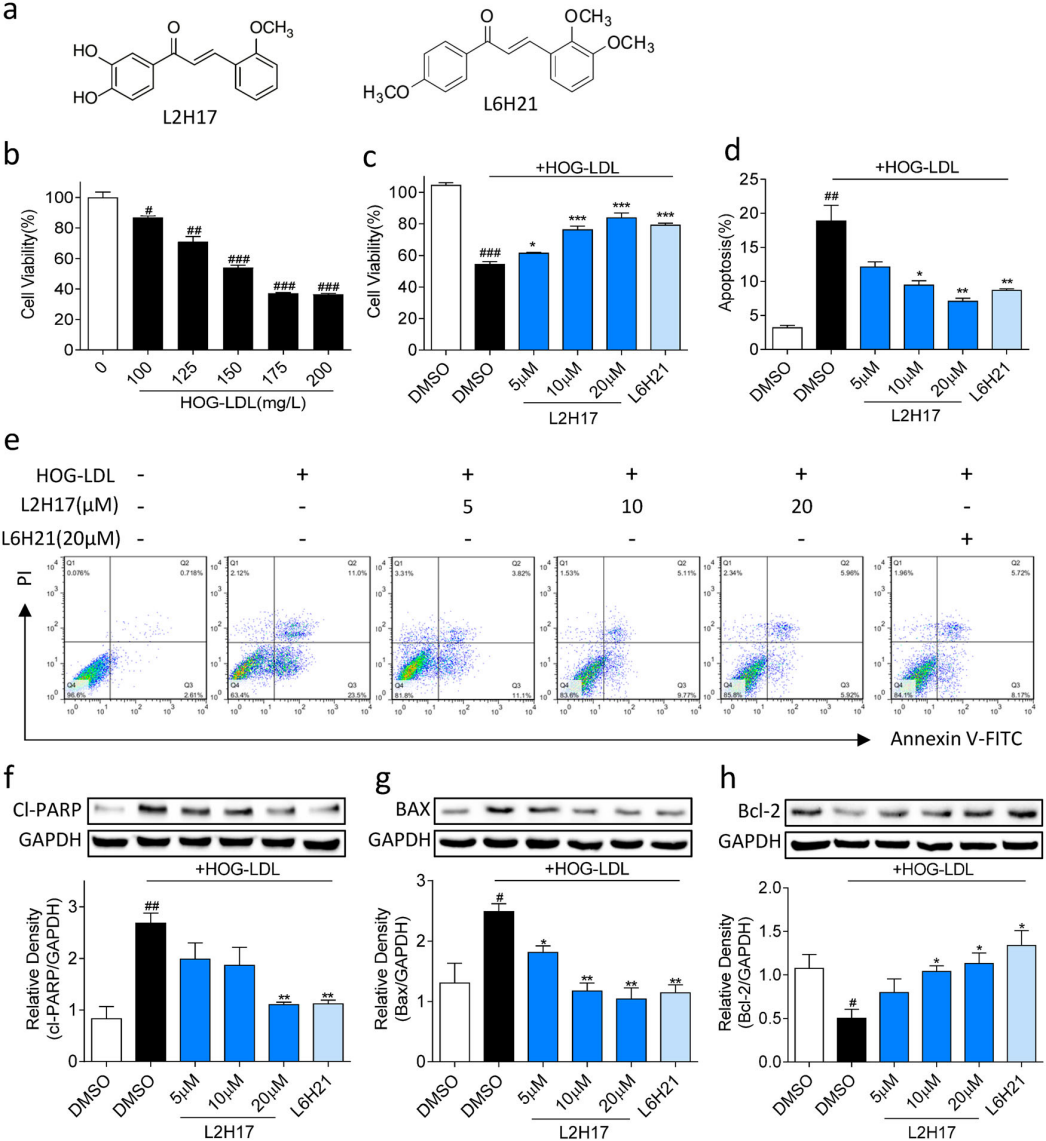
MD2 inhibitors reduce HOG-LDL-induced apoptotic cell death in MIO-M1 cells. **a** Chemical structures of L2H17 and L6H21. **b** The toxicity of HOG-LDL in MIO-M1 cells was measured 24 h after HOG-LDL treatment using the MTT assay. **c** Cells were pretreated with MD2 inhibitors for 2 h followed by challenge with HOG-LDL for 24 h. Cell viability was measured via the MTT assay. **d**, **e** MIO-M1 cells were pretreated with or without MD2 inhibitors for 12 h and then exposed to HOG-LDL. Apoptotic cells were detected by Annexin V/PI staining. Quantification of apoptotic cells (**d**) and (**e**) representative flow cytometry dot plots are shown. **f–h** Cells were treated as indicated in (**e**). The levels of cleaved PARP (**f**), Bax (**g**), and Bcl-2 (**h**) were determined using western blotting. GAPDH was used as the loading control. Quantitative data are presented as the mean ± SEM, *n* = 3; ^#^*P* < 0.05; ^##^*P* < 0.01; ^###^*P* < 0.001 compared to the DMSO control; *P < 0.05; **P < 0.01; ***P < 0.001 compared to HOG-LDL.

The chalcone analogs L2H17 and L6H21 were synthesized as described previously^23,27^. The compounds were structurally identified by MS and ^1^H NMR analyses. The chemical structures of L2H17 and L6H21 are shown in Fig. 1a. Both compounds were purified using HPLC and had a purity of 99%. L2H17 and L6H21 were dissolved in DMSO for in vitro studies and in 1% sodium carboxyl methyl cellulose (CMC-Na) for in vivo studies. HOG-LDL was purchased from Yiyuan Biotechnology (Guangzhou, China) and was prepared as described previously^28^. Apocynin, an inhibitor of NADPH oxidase^29^, was purchased from Sigma-Aldrich (St. Louis, MO). The reactive oxygen species probe 2′,7′-dichlorodihydrofluorescein diacetate (DCFH-DA) and the Micro Hydrogen Peroxide Assay kit (Catalog#: S0038) were purchased from Beyotime Biotech (Nantong, China). The One Step TUNEL Apoptosis Assay Kit was obtained from R&D Systems (Minneapolis, MN). Interleukin-1β (IL-1β) ELISA kits were obtained from eBioscience (San Diego, CA). Nuclear and cytosolic protein extraction kits were obtained from Trans AM Active Motif (Carlsbad, CA).

Primary antibodies against activating transcription factor-6α (ATF6α; sc-14253), B-cell lymphoma-2 (Bcl2; sc-23960), Bcl2-associated protein x (Bax; sc-23959), cleaved poly (ADP ribose) polymerase (PARP; sc-56196), glucose-regulated protein 78 (Grp78; sc-13539), inhibitor of κBα (IκBα; sc-371), myeloid differentiation primary response gene 88 (MyD88; sc-74532), myeloid differentiation protein 2 (MD2; sc-20668), nuclear factor-κB p65 subunit (p65; sc-372), NOX subunit p22^phox^ (sc-271968), Toll-like receptor 4 (TLR4; sc-10741), 8-hydroxydeoxyguanosine (8-OHdG; sc-393871), and GAPDH (sc-32233) and horseradish peroxidase-conjugated secondary antibodies were obtained from Santa Cruz Biotechnology (Santa Cruz, CA). Antibodies against activating transcription factor-4 (ATF4; 11815S), CCAAT-enhancer-binding protein homologous protein (CHOP; 2895S), and phosphorylated eukaryotic initiation factor-2α (p-eIF2α; 3398S) were purchased from Cell Signaling Technology (Beverly, MA). Alexa 647-conjugated secondary antibodies were obtained from Abcam (Cambridge, MA). The ApoB100 antibody was purchased from Proteintech (Rosemont, USA). Two different antibodies against NADPH oxidase-4 (NOX4; ab133303 and NB110–58849) were obtained from Abcam (Cambridge, MA) and Novus Biologicals (Centennial, CO), respectively.

The immortalized MIO-M1 human Müller cell line was obtained from the Institute of Ophthalmology and Moorfields Eye Hospital of London (London, United Kingdom). MIO-M1 cells were cultured in DMEM (Gibco, Eggenstein, Germany) containing 25 mmol/L D-glucose supplemented with 10% fetal bovine serum, 100 U/mL penicillin, and 100 mg/mL streptomycin. Cells were treated with compounds L2H17 and L6H21 in media for 2 h before exposure to HOG-LDL. DMSO was used as a vehicle control for HOG-LDL treatment. All experiments were repeated at least three times.

### Cell toxicity assays

MIO-M1 Müller cells were seeded in 96-well plates at a density of 1.0 × 10^4^/well and allowed to adhere overnight. In the initial studies, the cells were exposed to increasing concentrations of HOG-LDL to determine the ED_50_, which represents a 50% reduction in cell viability. Subsequent studies were carried out by exposing the cells to 150 mg/L HOG-LDL for 24 h, with or without pretreatment with MD2 inhibitors for 2 h. Cells were incubated with fresh growth media containing 5 mg/mL MTT for 4 h. The medium was removed, and the converted dye was solubilized with DMSO. The absorbance was measured at 490 nm. Cell viability is expressed as a percentage of the corresponding control.

To analyze apoptosis, cells were stained with Annexin V-FITC/PI, and the proportion of apoptotic cells was measured by a FACSCalibur (BD Biosciences, San Jose, CA). Briefly, MIO-M1 cells were exposed to 150 mg/L HOG-LDL for 12 h, with or without pretreatment with MD2 inhibitors for 2 h. Cells were then harvested and resuspended in 100 µL of 1× binding buffer, followed by the addition of 3 μL of Annexin V-FITC and 3 μL of PI. After the cells were incubated in the dark for 20 min, 400 μL of 1× binding buffer was added. The stained cells were mixed gently and subjected to flow cytometry. Analysis was performed using CellQuest software (BD Biosciences San Jose, CA) and FlowJo 7.6 software (TreeStar, San Carlos, CA).

### Immunoblotting

Protein concentrations in cell and tissue lysates were measured by Bradford assays (Bio-Rad laboratories, Hercules, CA). Samples were subjected to 12% sodium dodecyl sulfate polyacrylamide gel electrophoresis and transferred onto polyvinylidene fluoride membranes (Bio-Rad). The membranes were blocked with 5% nonfat milk in tris-buffered saline containing Tween (TBS-T) for 2 h at room temperature. The membranes were then incubated with primary antibodies at 4 °C overnight. The membranes were washed three times in TBS-T and incubated with appropriate horseradish peroxidase-conjugated secondary antibodies for 1 h at room temperature. Immunoreactivity was visualized using enhanced chemiluminescence reagents (Bio-Rad). Densitometric quantification was performed using ImageJ software (NIH, Bethesda, MD).

NF-κB p65 subunit levels and localization were determined by extracting nuclear and cytosolic fractions (Trans AM; Active Motif). GAPDH was used as a loading control for cytosolic proteins, and Lamin B was used as a loading control for nuclear proteins.

Interactions between MD2, TLR4, MyD88, and NOX4 were analyzed by immunoprecipitating the proteins with MD2 or TLR4 antibodies. Briefly, following treatments, extracts were prepared, incubated with MD2/TLR4 antibodies overnight at 4 °C, and immunoprecipitated with protein G-Sepharose beads at 4 °C for 2 h. The immunoprecipitation samples were immunoblotted to evaluate the coprecipitated proteins.

### Real-time quantitative PCR

MIO-M1 cells were treated with L2H17 at the indicated concentrations or 20 µM L6H21 for 2 h, followed by 150 mg/L HOG-LDL for 6 h. Total RNA was isolated from cells using TRIzol reagent (Invitrogen, Carlsbad, CA). Reverse transcription was performed using an M-MLV Platinum RT-qPCR Kit (Invitrogen). Real-time qPCR was carried out using a CFX96 Detection System (Bio-Rad). The primer sequences for TNF-α, IL-6, IL-1β, and β-actin are listed in Supplementary Table S1. The relative amount of each target transcript was normalized to β-actin.

### MD2 and NOX4 silencing

Cells were plated in normal growth media without antibiotics at a density of 5 × 10^4^ cells per well overnight. The cells were then transfected with siRNA against MD2 and NOX4 using Lipofectamine 2000 (Invitrogen). The siRNA sequences for MD2, NOX4, and the negative control are listed in Supplementary Table S2. A final concentration of 100 nM siRNA was used. The medium was replaced with complete growth media after 6 h. Cells were cultured for and additional 24 h before the knockdown efficiency was determined and treatments were initiated.

### Immunofluorescence cell labeling

MIO-M1 cells were seeded on 15 mm glass bottom cell culture dishes and allowed to attach overnight. The cells were treated with 20 µM L2H17 or L6H21 for 2 h and then exposed to 150 mg/L HOG-LDL for 3 h. The cells were fixed with paraformaldehyde and permeabilized with Triton X-100. ATF6α primary antibodies were added and incubated at 4 °C overnight. Immunoreactivity was detected by Alexa-647-conjugated secondary antibodies. The cells were counterstained with DAPI. Fluorescence images were captured using a Nikon epifluorescence microscope (Nikon, Tokyo, Japan).

The levels of ROS in cells were also determined by labeling the cells with the ROS probe DCFH-DA, as described previously^30^. The cells were treated as described for ATF6α staining and then incubated with 2 µM DCFH-DA. The fluorescence intensity of 8000 events was acquired using a FACSCalibur flow cytometer (BD Biosciences). Analysis was performed using CellQuest software (BD Biosciences) and FlowJo 7.6 software (TreeStar, San Carlos, CA). Images of the labeled cells were also captured using an epifluorescence microscope.

### Mouse model of diabetes

All animal studies were approved by the Wenzhou Medical University Animal Policy and Welfare Committee (Approved documents: wydw2014–0058). Male C57/BL6 mice weighing 18–22 g and at 4–6 weeks of age were obtained from the Animal Center of Wenzhou Medical University. The mice were divided into control and diabetic groups. Control mice were maintained on a standard diet containing 10 kcal.% fat, 20 kcal.% protein, and 70 kcal.% carbohydrate (Cat. #MD12031, HFK Bioscience Co. Ltd). Mice in the diabetic group were fed a high-fat diet containing 60 kcal.% fat, 20 kcal.% protein, and 20 kcal.% carbohydrate (Cat. #MD12033; HFK Bioscience Co. Ltd). This diet was administered for 30 weeks. Mice that were fed with the high-fat diet received a single intraperitoneal injection of 50 mg/kg streptozotocin (STZ; Sigma-Aldrich) at week 10 and another injection of 80 mg/kg STZ at week 11. STZ was dissolved in 100 mM citrate buffer (pH 4.5). Mice in the control group (standard diet) received citrate buffer alone. Mice with fasting blood glucose levels greater than 250 mg/dL were considered diabetic. L2H17 (20 mg/kg) was administered by oral gavage every two days beginning at week 26. Untreated diabetic mice and nondiabetic control mice received the CMC-Na vehicle only. Each experimental group (control, diabetic mice, and diabetic mice treated with L2H17) included at least eight mice. Body weights and blood glucose levels were measured weekly.

### Scotopic electroretinogram (ERG) recordings

The mice were dark-adapted for 4 h and then prepared under dim red light. The mice were anesthetized using 4% chloral hydrate, and their pupils were dilated using 0.5% tropicamide with topical 0.5% proxymetacaine HCl. Standard ERG recordings were made for both eyes of each mouse with corneal electrodes. The reference electrodes were subcutaneously cannulated into a visor, and a stainless-steel sheet under the animals was used as the ground electrode. Each stimulus (optical power 0 cds/m^2^, bandpass filtered from 1 to 300 Hz) was given once to every responder with a duration of 250 ms. Amplitudes were measured and recorded for both a- and b-waves by RETI2 scan vision electrophysiology detection (Roland Consult, Brandenburg, Germany). The average amplitudes of a- and b-waves and op amplitudes were calculated. The amplitude of the a-wave was measured from the baseline to the bottom of the a-wave, and the amplitude of the b-wave was measured from the bottom of the a-wave to the peak of the b-wave, as described previously^31^.

### Tissue collection and analyses

Four weeks after initiating L2H17 treatment, the mice were euthanized. Blood samples were collected for serum lipid profiling. Total triglycerides (TGs), total cholesterol (TCH), low-density lipoprotein (LDL), and high-density lipoprotein (HDL) were measured by assay kits obtained from Nanjing Jiancheng (Jiangsu, China). Both eyes were removed. Samples were embedded in optimal cutting media for immunofluorescence analysis of 8-OHdG and ApoB100. For TUNEL staining, samples were fixed in 4% paraformaldehyde and embedded in paraffin. Samples for immunoblotting and IL-1β ELISA were snap-frozen.

TUNEL staining was carried out on 6 µm-thick paraffin sections. The slides were dewaxed, hydrated, treated with proteinase K, and permeabilized using Triton X-100. TUNEL staining solutions were then added. Tissues were counterstained with DAPI, and the slides were preserved using fluorescence mounting media. For immunofluorescence detection of 8-OHdG and ApoB100, 6 μm-thick frozen sections were used. The slides were incubated with antibodies against 8-OHdG or ApoB100 (1:200) at 4 °C overnight. Alexa-647-conjugated secondary antibodies were used for detection. The numbers of TUNEL-, 8-OHdG-, and ApoB100-positive cells were counted.

### Statistical analysis

The data are presented as the mean ± SEM. The exact group size (*n*) for each experimental group/condition is provided, and “*n*” refers to independent values, not replicates. Statistical analysis was performed with GraphPad Prism 6.0 software (San Diego, CA, USA). We used one-way ANOVA followed by Dunnett’s post hoc test when comparing more than two groups of data. Differences were considered significant at *P* < 0.05.

## Results

### MD2 inhibitors reduce HOG-LDL-induced apoptosis in retinal Müller cells

Müller cells contribute to the pathogenesis of diabetic retinopathy by altering the release of growth factors and proinflammatory cytokines^32^. Studies also indicate loss of Müller cells as diabetic retinopathy progresses^32^. Therefore, we examined the effect of highly oxidized glycated LDL (HOG-LDL) on the human Müller cell line MIO-M1. Exposure to increasing concentrations of HOG-LDL reduced the viability of MIO-M1 cells, and a 50% decrease was observed at 150 mg/L (Fig. 1b). These results are consistent with previous studies showing a 50% reduction in the viability of Müller cells in response to 100–200 mg protein/L HOG-LDL^10^. Pretreatment with both MD2 inhibitors abrogated the HOG-LDL-induced decrease in viability (Fig. 1c). Annexin V/PI staining of cells showed that the MD2 inhibitors prevented HOG-LDL-induced apoptotic death in MIO-M1 cells (Fig. 1d, e). These protective effects of the MD2 inhibitors were reflected in the normalization of HOG-LDL-induced levels of the caspase substrate Cl-PARP and apoptosis-related protein Bax (Fig. 1f, g). The MD2 inhibitors also increased Bcl2 levels in MIO-M1 cells exposed to HOG-LDL (Fig. 1h). These results show that MD2 inhibitors rescue Müller cells from HOG-LDL-induced toxicity and suggest a role of MD2 in Müller cell dysfunction in diabetic retinopathy.

### MD2 inhibitors prevent HOG-LDL-induced inflammatory responses in Müller cells

**Fig. 2:**
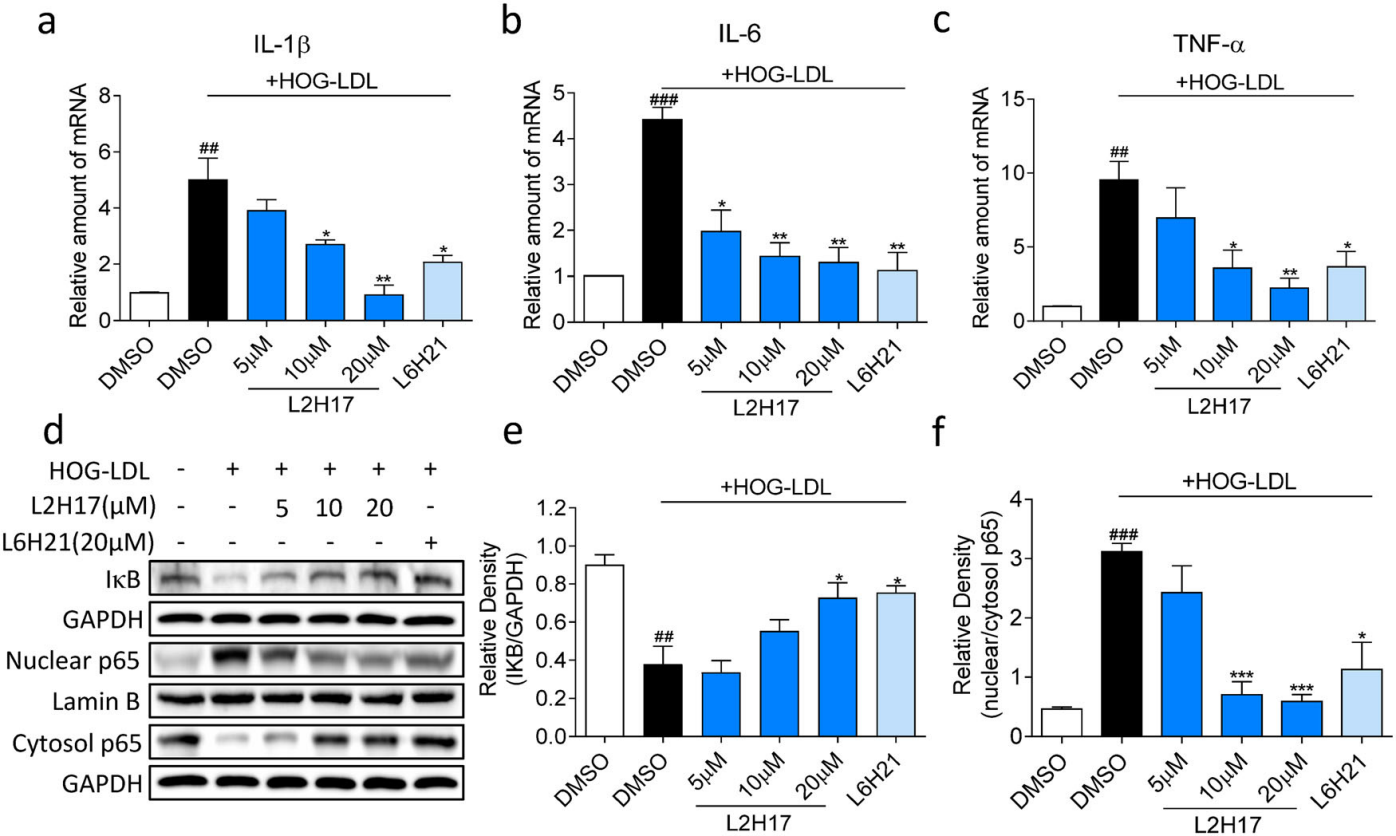
MD2 inhibitors suppress HOG-LDL-induced inflammatory signaling in MIO-M1 cells. MIO-M1 cells were pretreated with MD2 inhibitors for 2 h, followed by HOG-LDL exposure. **a**–**c** The mRNA levels of IL-1β (**a**), IL-6 (**b**), and TNF-α (**c**) were determined 6 h after HOG-LDL stimulation using RT-qPCR. **d** Western blot analysis of cytosolic IκBα, nuclear NF-κB subunit p65, and cytosolic p65 in MIO-M1 cells was evaluated 2 h after HOG-LDL exposure. GAPDH was used as a loading control for cytosolic proteins, and lamin B was used as a loading control for nuclear proteins. Representative blots of three independent experiments are shown. Densitometric quantification of IκBα levels (**e**) and nuclear/cytosolic p65 ratios (**f**) are shown. Quantitative data are presented as the mean ± SEM, *n* = 3; ^##^*P* < 0.01; ^###^*P* < 0.001 compared to the DMSO control; *P < 0.05; **P < 0.01; ***P < 0.001 compared to HOG-LDL.

Since Müller cells produce proinflammatory cytokines in the context of diabetes, we determined whether MD2 inhibition altered HOG-LDL-induced inflammatory responses in cells. We measured the transcriptional levels of IL-1β, IL-6, and TNF-α in MIO-M1 cells challenged with HOG-LDL, with or without pretreatment with MD2 inhibitors. HOG-LDL induced the expression of inflammatory cytokines by 4~9-fold (Fig. 2a–c). Treatment with MD2 inhibitors completely prevented these inductions. Since MD2 is essential for TLR4 signaling through nuclear factor-κB (NF-κB) and all three examined inflammatory cytokines in MIO-M1 cells have a NF-κB regulatory element, we assessed NF-κB activity by measuring the levels of inhibitor of κBα (IκBα) and the nuclear translocation of the p65 subunit of NF-κB. Our results showed that HOG-LDL reduced the levels of IκBα and increased the nuclear translocation of p65, signifying enhanced NF-κB activity (Fig. 2d–f). However, both L2H17 and L6H21 markedly reversed these effects, suggesting that MD2 inhibitors prevented HOG-LDL-induced NF-κB activation and downstream inflammatory cytokine production.

**Fig. 3:**
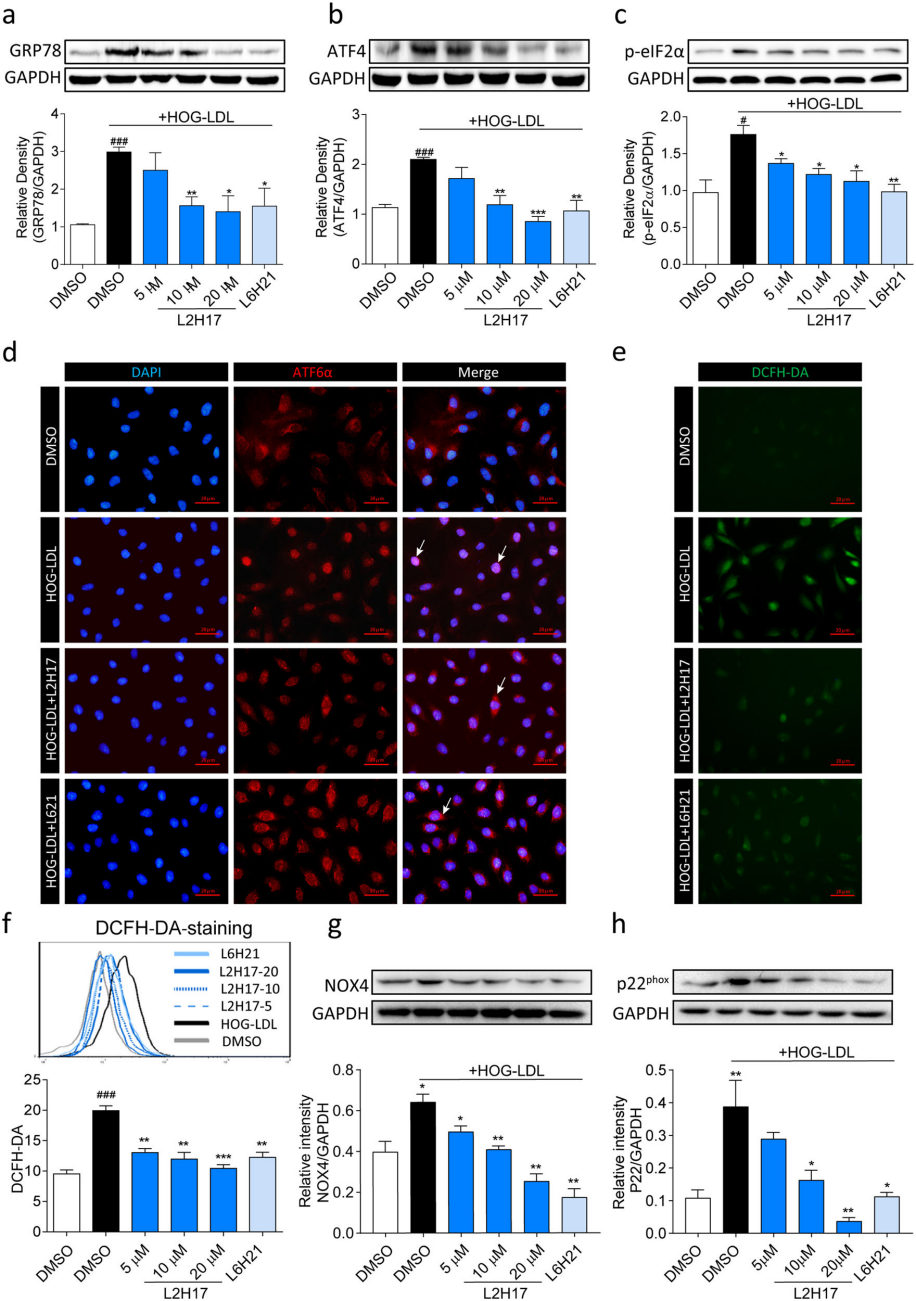
MD2 inhibitors attenuate HOG-LDL-induced ER stress and ROS generation in MIO-M1 cells. **a–c** MIO-M1 cells were pretreated with or without MD2 inhibitors and exposed to HOG-LDL for 12 h, and ER stress was evaluated by determining the levels of GRP78 (**a**), ATF4 (**b**), and p-eIF2α (**c**). GAPDH was used as the loading control. **d** Cells were exposed to HOG-LDL for 3 h, and the subcellular localization of ATF6α (red) was determined by immunofluorescence staining. Cells were counterstained with DAPI (blue) (scale bar = 20 µm). **e**, **f** Cells were treated as described in (**d**) and stained with the ROS probe DCFH-DA. Fluorescence images (**e**) were captured (scale bar = 20 µm). The quantification of ROS levels was performed by flow cytometry (**f**). **g**, **h** The levels of NOX4 (**g**) and NOX4 subunit p22^Phox^ (**h**) were measured in total lysates. Densitometric quantification was performed by normalizing the levels to GAPDH. Quantitative data are presented as the mean ± SEM, *n* = 3; ^#^*P* < 0.05; ^##^*P* < 0.01; ^###^*P* < 0.001 compared to the DMSO control; *P < 0.05; **P < 0.01; ***P < 0.001 compared to HOG-LDL.

An important mechanism by which modified LDL, including HOG-LDL, causes cytotoxicity in Müller cells is the induction of oxidative stress and endoplasmic reticulum (ER) stress^10^. Therefore, we assessed whether the MD2 inhibitors, which blunted HOG-LDL-induced inflammatory responses and apoptosis, also normalized ER stress and oxidative stress. For ER stress assessment, we measured related proteins, including GRP78, ATF4, and p-eIF2α. We showed that the levels of all three ER stress-related proteins were elevated after 12 h of exposure to HOG-LDL (Fig. 3a–c). Pretreatment of the cells with L2H17 and L6H21 prevented the increases in these ER stress markers. We further examined the nuclear translocation of ATF6α and observed increased nuclear immunoreactivity of ATF6α, which was indicated by ATF6α and DAPI double-positive staining, in HOG-LDL-challenged cells, but ATF6α nuclear translocation was reduced in cells that were pretreated with L2H17 and L6H21 (Fig. 3d). These two approaches show that MD2 inhibitors reduce HOG-LDL-induced ER stress pathway activation in Müller cells.

Next, we examined the levels of reactive oxygen species (ROS), as ER stress and oxidative stress are closely related in most cell types. Here, we showed that HOG-LDL rapidly increased cellular ROS levels and that these levels were significantly reduced by L2H17 and L6H21 pretreatment (Fig. 3e, f). Additionally, HOG-LDL significantly increased the level of H_2_O_2_ in MIO-M1 cells (Supplementary Fig. S1). However, the HOG-LDL-dependent increase in H_2_O_2_ was reversed by MD2 inhibitors (Supplementary Fig. S1). In support of these results, we observed increased levels of NADPH oxidase-4 (NOX4) and the p22^Phox^ subunit of NOX4 when cells were challenged with HOG-LDL (Fig. 3g, h). As expected, L2H17 and L6H21 significantly inhibited the levels of NOX4 and p22^Phox^ (Fig. 3g, h). These results suggest that NOX4 may be a source of elevated ROS in response to HOG-LDL.

### MD2 mediates HOG-LDL-induced activation of the TLR4 pathway and TLR4-NOX4 interaction

**Fig. 4:**
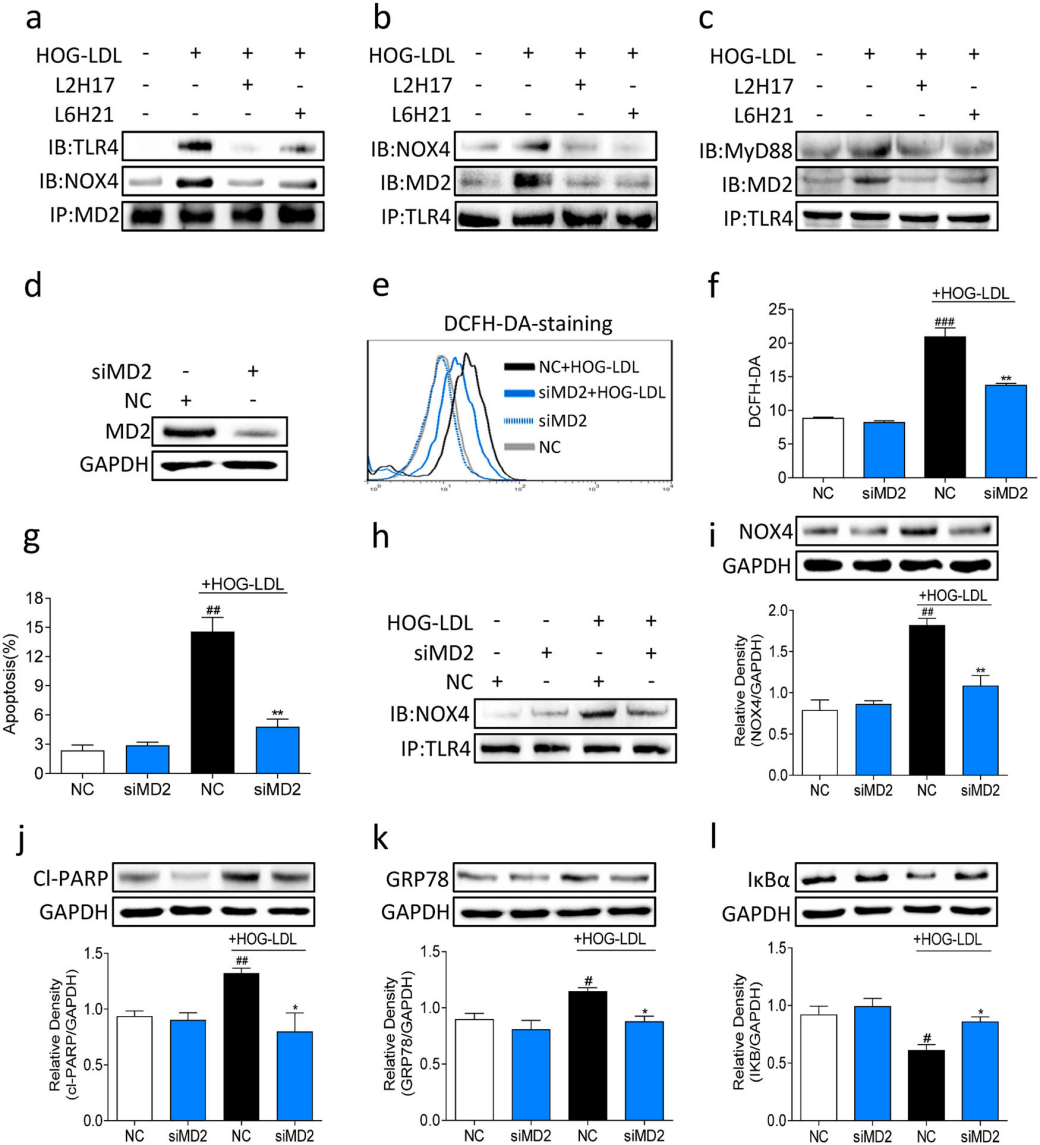
HOG-LDL causes MIO-M1 cell apoptosis by enhancing the NOX4-TLR4 interaction in a MD2-dependent manner. **a–c** MIO-M1 cells were pretreated with or without MD2 inhibitors for 2 h and exposed to HOG-LDL for 15 min. Protein samples were immunoprecipitated with MD2 antibodies (**a**) or TLR4 antibodies (**b**, **c**). Associated proteins were detected using immunoblotting. **d** MIO-M1 cells were transfected with siRNA against MD2 (siMD2) or negative control (NC) siRNA. The levels of MD2 were measured after 24 h. GAPDH was used as a loading control. **e**, **f** Cells transfected with siMD2 or NC were exposed to HOG-LDL for 3 h, and ROS levels were measured by DCFH-DA. **e** Shows representative flow cytometry data. The quantification of ROS levels is shown in (**f**). **g** MIO-M1 cells were transfected with siMD2 or NC and exposed to HOG-LDL for 12 h. The number of apoptotic cells was determined using flow cytometry. **h** Cells were transfected with siMD2 or NC and exposed to HOG-LDL for 15 min. Protein samples were immunoprecipitated with TLR4 antibody, and NOX4 was detected by immunoblotting. **i–l** Cells were transfected with siMD2 or NC and exposed to HOG-LDL for 12 h to analyze NOX4 (**i**), cleaved PARP (**j**), and GRP78 (**k**) or 2 h to analyze IκBα (**l**). GAPDH was used as a loading control. Densitometric quantification is shown in the lower panels. Quantitative data are presented as the mean ± SEM, *n* = 3; ^#^*P* < 0.05; ^##^*P* < 0.01; ^###^*P* < 0.001 compared to the untreated NC; *P < 0.05; **P < 0.01 compared to the HOG-LDL-treated NC.

It has been shown that lipopolysaccharide (LPS)-induced ROS generation and NF-κB activation are mediated by a direct interaction between TLR4 and NOX4^33^. LPS is the prototypical ligand of TLR4 and requires MD2 for TLR4 activation. These studies raise the exciting possibility that HOG-LDL-induced ROS levels and NF-κB activation in Müller cells is also mediated through MD2/TLR4 activation and the interaction between TLR4 and NOX4. To test this hypothesis, we challenged MIO-M1 cells with HOG-LDL, with or without pretreatment with MD2 inhibitors. MD2 immunoprecipitation showed an association between TLR4 and NOX4 in HOG-LDL-exposed cells (Fig. 4a). This complex formation was dramatically reduced in cells that were treated with MD2 inhibitors prior to HOG-LDL exposure. Similar results were obtained when we immunoprecipitated TLR4 and probed for MD2 and NOX4 (Fig. 4b). Additionally, we assessed the interaction between TLR4 and MyD88, which is a key TLR4 adaptor protein that mediates NF-κB activation in response to TLR4 activation. Our results showed that HOG-LDL increased the TLR4-MyD88 interaction (Fig. 4c), and as expected, this interaction was significantly reduced in cells that were treated with MD2 inhibitors.

To confirm the key effects of MD2 inhibitors on Müller cells, we transfected the cells with siRNA targeting MD2 (Fig. 4d). MD2 silencing significantly attenuated HOG-LDL-induced ROS levels (Fig. 4e, f) and the number of apoptotic cells (Fig. 4g). We then exposed MD2 siRNA-transfected cells to HOG-LDL and showed reduced TLR4-NOX4 interaction (Fig. 4h) and reduced NOX4 protein levels (Fig. 4i). In addition, MD2 silencing reduced the levels of cleaved PARP (Fig. 4j), GRP78 (Fig. 4k), and IκBα (Fig. 4l) in cells exposed to HOG-LDL. These results confirm the data obtained by L2H17 and L6H21 treatment of cells and showed that MD2 played a role in mediating HOG-LDL-induced activation of the TLR4-NOX4 signaling pathway.

**Fig. 5:**
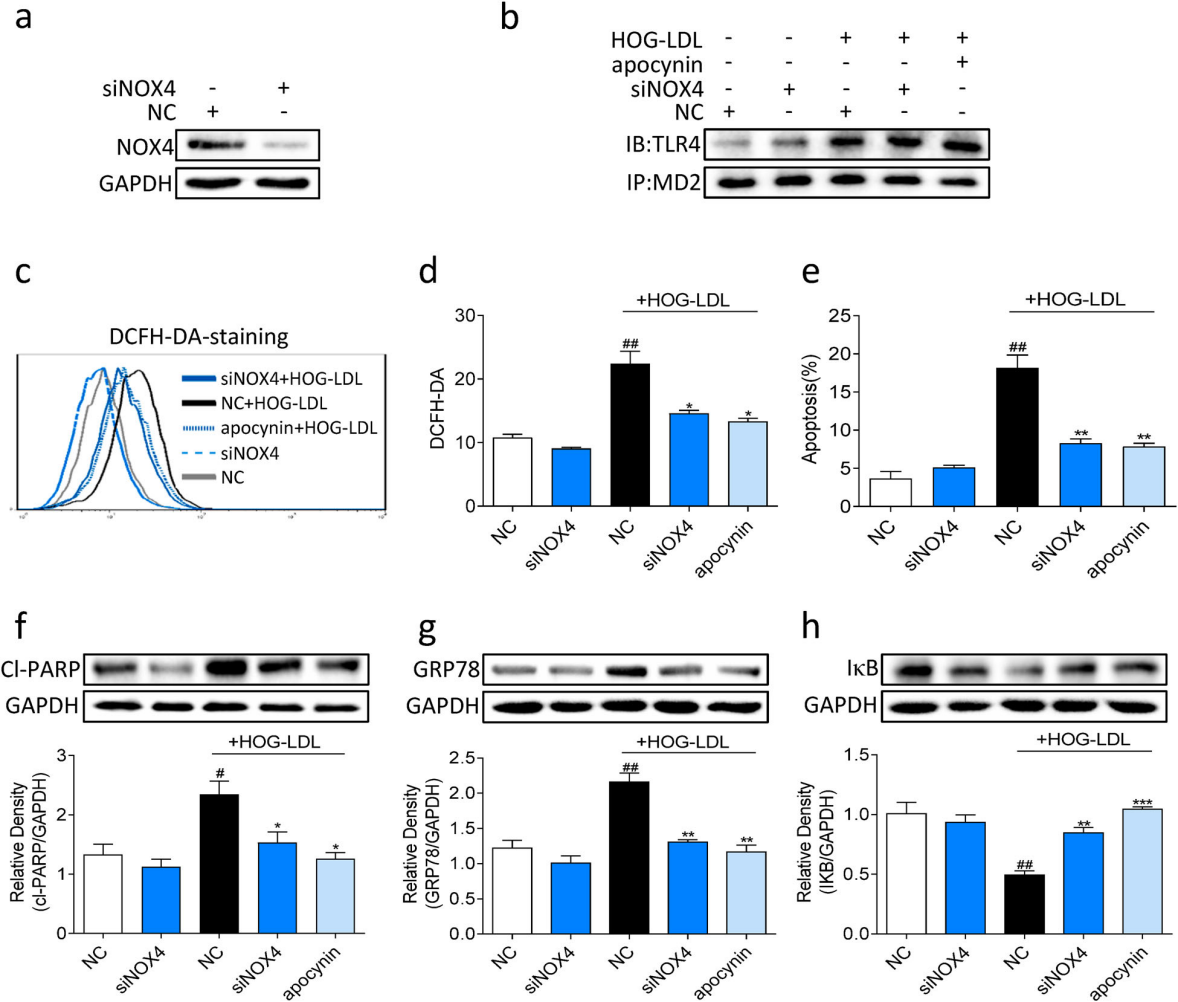
NOX4 acts as a downstream signaling component of the HOG-LDL-induced TLR4/MD2 pathway. **a** MIO-M1 cells were transfected with siRNA against NOX4 (siNOX4) or NC. The levels of NOX4 were determined after 24 h by immunoblotting. GAPDH was used as a loading control. **b** MIO-M1 cells were transfected with siNOX4 or NC and exposed to HOG-LDL for 15 min with or without pretreatment with apocynin. The effect of NOX4 silencing on the MD2-TLR4 interaction was determined by immunoprecipitating the protein samples with a MD2 antibody. **c**, **d** MIO-M1 cells were transfected with siNOX4 or NC and exposed to HOG-LDL for 3 h with or without pretreatment with apocynin for 2 h. ROS levels were determined using flow cytometry. **e** MIO-M1 cells were transfected with siNOX4 or NC and exposed to HOG-LDL for 12 h with or without pretreatment with apocynin for 2 h. The number of apoptotic cells was determined by Annexin V/PI staining and flow cytometry. **f–h** Cells were transfected with siNOX4 or NC and exposed to HOG-LDL with or without pretreatment with apocynin for 12 h to analyze cleaved PARP (**f**) and GRP78 (**g**) or 2 h to analyze IκBα (**h**). GAPDH was used as a loading control. Densitometric quantification is shown in the lower panels. Quantitative data are presented as the mean ± SEM, *n* = 3; ^#^*P* < 0.05; ^##^*P* < 0.01, compared to the untreated NC; *P < 0.05; **P < 0.01; ***P < 0.001 compared to the HOG-LDL-treated NC.

To directly determine whether the NOX4 interaction with TLR4 is important for ROS generation and the downstream activity of TLR4, we silenced NOX4 in MIO-M1 cells (Fig. 5a). For these experiments, we also used apocynin, which acts as an inhibitor of NOX, as the positive control. NOX4 depletion and pharmacological inhibition failed to alter the MD2-TLR4 interaction in cells exposed to HOG-LDL (Fig. 5b). However, silencing NOX4 or inhibiting NOX activity by apocynin prevented HOG-LDL-induced ROS generation (Fig. 5c, d) and MIO-M1 cell apoptosis (Fig. 5e). In addition, Cl-PARP levels were suppressed in cells transfected with NOX4 siRNA and cells pretreated with apocynin (Fig. 5f), consistent with a reduction in apoptotic cell death. The levels of GRP78 were also suppressed upon NOX4 deficiency (Fig. 5g), which was consistent with suppressed ROS generation. However, we observed increased IκBα levels in NOX4 siRNA-transfected cells exposed to HOG-LDL (Fig. 5h). These results suggest that the NOX4 interaction with TLR4 is essential for NF-κB activation by HOG-LDL.

### MD2 inhibition protects against diabetes-induced retinal dysfunction

Our final objective was to determine whether inhibiting MD2 in an animal model of diabetes would prevent or alter the progression of diabetes-induced retinal injuries. We used a combined high-fat diet (HFD)/streptozotocin (STZ) mouse model of type 2 diabetes. Weekly body weight measurements showed dramatic weight gain in mice maintained on a HFD (Supplementary Fig. S2a). Although STZ administration slightly reduced the body weights, the results were not significantly different compared to the pre-STZ state. Treatment of diabetic mice with L2H17 also did not alter body weight gain in the mice. Blood glucose levels were increased following STZ injection (Supplementary Fig. S2b). No changes in blood glucose levels were noted in mice treated with L2H17 compared to untreated diabetic mice. Similarly, serum levels of TCH, TGs, LDL, and HDLs were not altered by L2H17 (Supplementary Fig. S2c–f).

**Fig. 6:**
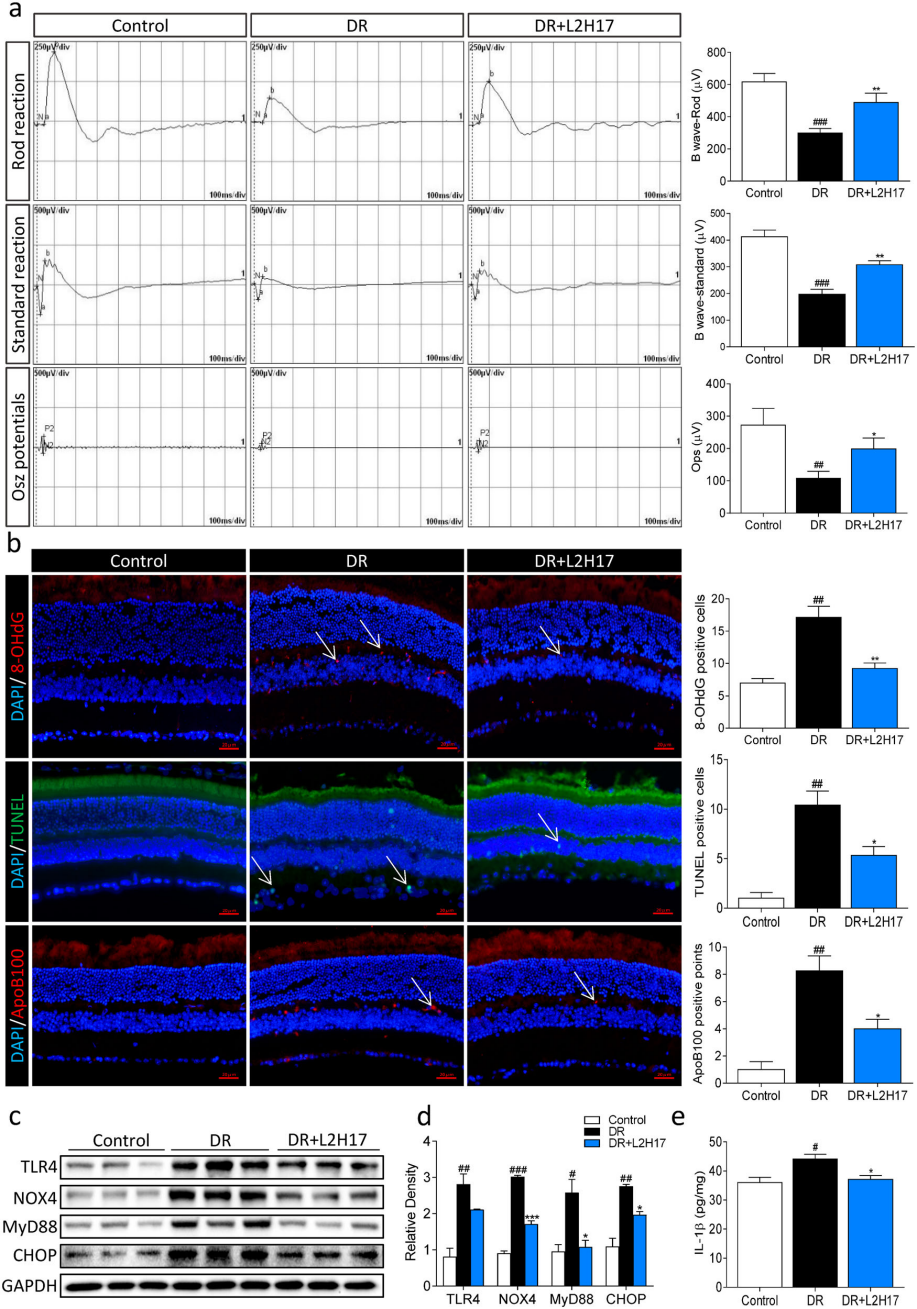
L2H17 reduces diabetes-induced retinal dysfunction. **a** Mouse retinal function was determined by electroretinography. Representative electroretinograms (left) and quantification (right) showing the Rod reaction, Max reaction, and Osz potentials (Ops). **b** Immunofluorescence labeling of 8-OHdG (red), TUNEL (green), and ApoB100 (red) in retinal sections. The number of positive cells (arrow) was counted (Scale bar = 20 μm). **c**, **d** The levels of TLR4, NOX4, MyD88, and the ER stress-related protein CHOP in the retinal tissues of mice. GAPDH was used as a loading control. Representative blots are shown in (**c**), and densitometric quantification is shown in (**d**). **e** IL-1β protein levels in retinal tissue lysates were measured by ELISA. The data were normalized to the total amount of proteins in the samples. Quantitative data are presented as the mean ± SEM, *n* = 4–8; ^#^*P* < 0.05; ^##^*P* < 0.01; ^###^*P* < 0.001 compared to nondiabetic control mice; *P < 0.05; **P < 0.01; ***P < 0.001 compared to untreated diabetic mice.

To assess retinal function in diabetic mice, we used noninvasive electroretinography^34,35^. Previous studies have shown that one of the earliest signs of diabetic retinopathy is a prolongation of the implicit time or a reduction in the amplitude of oscillatory potentials (Ops)^36^. In addition, a delayed peak time and reduced b-wave amplitude are highly correlated with the severity of diabetic retinopathy^35,37,38^. In our study, we noted significantly reduced b-wave amplitudes of the Rod reaction and Max reaction and Ops amplitude in diabetic mice compared to the nondiabetic controls (Fig. 6a). However, these reductions were attenuated in diabetic mice treated with the MD2 inhibitor L2H17 (Fig. 6a). As b-waves and Ops mainly reflect the function of the inner retina, which is composed of ON-bipolar cells and Müller cells, our results suggest that L2H17 had a protective effect on Müller cells and the inner retinal functions of diabetic mice.

We next evaluated whether MD2 inhibition prevented oxidative stress and inflammatory responses in the retinas of diabetic mice. To measure oxidative stress, we performed immunofluorescence staining of retinal tissues for 8-hydroxy-2′-deoxyguanosine (8-OHdG), which is a predominant oxidative DNA lesion. The levels of 8-OHdG immunoreactivity were increased in the retinas of diabetic mice (Fig. 6b, upper row). This positivity was primarily localized to the inner retina, which was consistent with the electroretinography results. In addition, apoptotic cell death, as detected by TUNEL staining, was observed in the inner retinal cells of diabetic mice (Fig. 6b, middle row). Treatment of diabetic mice with L2H17 significantly reduced 8-OHdG staining and the number of TUNEL-positive cells. We then determined whether these deleterious changes in the retina in diabetic mice were associated with modified LDL. Since ox-LDL may include numerous epitopes, we examined the levels of LDL-predominant apolipoprotein B100 (ApoB100). At a minimum, this readout would show LDL extravasation. Stained retinal tissues showed increased ApoB100 immunoreactivity in the inner retina (Fig. 6b, lower row). Surprisingly, L2H17 treatment reduced ApoB100 reactivity in the retinal tissues of diabetic mice.

**Fig. 7:**
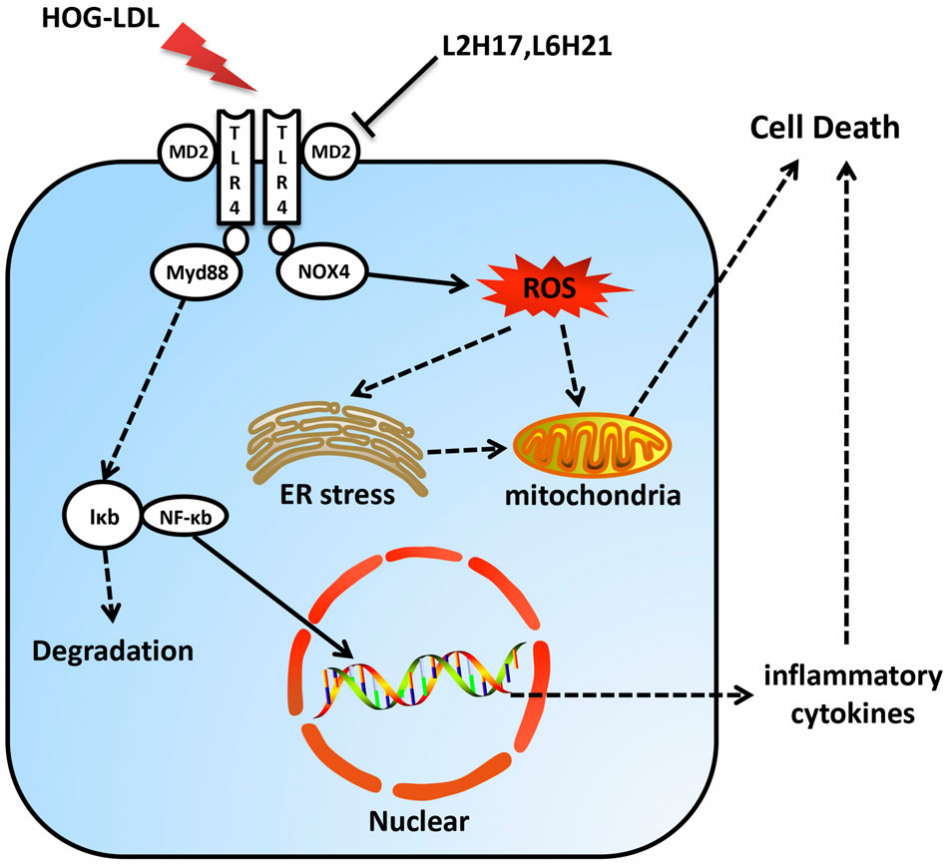
Proposed mechanism for TLR4/MD2 signaling and the effects of MD2 inhibitors on HOG-LDL-induced Müller cell dysfunction. HOG-LDL activates TLR4 through MD2 in Müller cells. TLR4 activation is associated with the NOX4 and MyD88 interaction. The MyD88 pathway activates NF-κB to induce the expression of proinflammatory cytokines. However, the NOX4-TLR4 interaction increases ROS levels, leading to ER stress and mitochondrial dysfunction. Oxidative stress and unregulated inflammatory responses cause Müller cell apoptosis. Chalcone inhibitors of MD2 disrupt MD2-TLR4-NOX4 complex formation in HOG-LDL-challenged cells. The suppression of downstream oxidative stress and normalization of inflammatory responses prevent retinal Müller cell death.

The preservation of retinal function and retinal cell integrity by L2H17 suggested that MD2 inhibition could suppress inflammatory responses in diabetic mice. The analysis of retinal tissue lysates showed increased levels of TLR4, MyD88, and NOX4 in diabetic mice (Fig. 6c, d). As expected, MD2 inhibition prevented the increases in NOX4 and MyD88. However, no significant changes in TLR4 levels were observed upon MD2 inhibition. CHOP, which is induced by ER stress and mediates apoptosis, was examined and showed that MD2 inhibition prevented diabetes-induced ER stress in the retina (Fig. 6c, d). In full agreement with these results, the levels of the inflammatory cytokine IL-1β were suppressed by MD2 inhibition in diabetic mice (Fig. 6e). In summary, the treatment of diabetic mice with a MD2 inhibitor reduced LDL extravasation into the retina, reduced oxidative and ER stress, and suppressed inflammatory responses (Fig. 7).

## Discussion

In this study, we showed that the inhibition of MD2 significantly reduced HOG-LDL-induced inflammatory signaling and oxidative stress in retinal Müller cells. This reduction was evidenced by reduced apoptosis following HOG-LDL challenge. We also showed that the mechanisms underlying the adverse effects of HOG-LDL on Müller cells included activation of the TLR4-MyD88 and TLR4-NOX4 signaling pathways. Inhibition of MD2 reduced TLR4-MyD88 complex formation, suppressed NF-κB activity, and prevented HOG-LDL-induced proinflammatory cytokine expression. MD2 inhibition also reduced the interaction between TLR4 and NOX4, resulting in decreased ROS generation. Finally, we showed that MD2 inhibition prevented diabetes-induced retinal dysfunction, oxidative stress and cellular apoptosis. Collectively, these findings provide support for a key role of MD2 in mediating diabetes/hyperlipidemia-induced Müller cell injury. In addition, the results point to MD2 as a potential target for the treatment of diabetic retinopathy.

Several studies have linked TLR4 to a potential inflammatory response in diabetic retinopathy^39,40^. The levels of TLR4 and downstream signaling proteins in the TLR4 signaling pathway have been shown to be increased in cultured cells exposed to high levels of glucose^39–42^, in the fibrovascular membranes of patients with diabetic retinopathy^40^, and in STZ-induced experimental models of diabetes^40^. These increases are associated with upregulated inflammatory cytokines^39,41,42^ and are believed to be induced by the TLR4 ligand HMGB-1^42^. Inhibiting TLR4 through TAK-242 decreases TLR4, TLR4 downstream signaling molecules, and proinflammatory cytokines in STZ-induced diabetic rats^19^. Consistent with these results, we showed that diabetes increased TLR4 and MyD88 levels in retinal tissues. Similar to direct TLR4 inhibition^19^, we showed that disrupting MD2 prevented diabetes-induced inflammatory responses in the retina. However, unlike the previous study^19^, MD2 inhibition did not reduce TLR4 levels. This effect is possibly due to feedback and the nature of inhibition. Whether it is therapeutically beneficial to preserve TLR4 levels while inhibiting the downstream inflammatory signaling remains to be determined.

Increasing evidence also indicates that modified LDL may play a role in the progression of diabetic retinopathy^8,43^. Although a recent study reported that activation of AMP-activated protein kinase (AMPK) protects against HOG-LDL-induced cell injury^44^, the exact mechanisms by which modified LDL causes cell injury are not fully understood. Our study is the first to address the role of MD2 in modified LDL-induced toxicity in retinal cells. In cultured Müller cells, we showed that HOG-LDL increased ROS levels, leading to ER stress and apoptosis. The induction of ROS in these retinal cells required MD2, since pharmacological inhibition and silencing of MD2 suppressed ROS levels. However, there remains a knowledge gap as to exactly how MD2, the TLR4 coreceptor, regulates HOG-LDL-induced ROS production. Recent studies have suggested that NOX4 may interact with TLR4 to generate ROS under inflammatory^45^ and hypoxic conditions^46^. Moreover, NOX4 inhibition exerts protective effects on the retinas of db/db diabetic mice^47,48^. These findings suggest that MD2 may represent a link between HOG-LDL, TLR4 and NOX4. Our results certainly support this conclusion. We showed that MD2 inhibition significantly reduced the interaction between TLR4 and NOX4 upon the exposure of Müller cells to HOG-LDL. Suppression of TLR4-NOX4 complex formation was associated with reduced ROS levels in cells. Furthermore, the effect of MD2 inhibition could be mimicked by the NOX inhibitor apocynin. This finding suggests that MD2 plays an important role in mediating HOG-LDL-induced NOX activation by facilitating the TLR4-NOX4 interaction. This finding also raises an interesting question as to whether the TLR4-MyD88 and TLR4-NOX4 pathways are truly distinct. The results of our study suggest that they are not or that there is significant cross-talk between the two pathways at a minimum. For example, NOX4 silencing or inhibition through apocynin completely reversed IκBα levels in HOG-LDL-exposed cells. It is possible that this reversal occurs through the suppression of ROS. However, this result may also indicate that NOX4 inhibition prevented TLR4-MyD88 activation. These possibilities should be tested in future studies.

An interesting finding of our study was that MD2 inhibition reduced ApoB100 immunoreactivity in the retinas of diabetic mice. These results suggest that MD2 inhibition reduces LDL extravasation and/or retention. Although this hypothesis needs to be further tested, it is possible that MD2 inhibition in retinal vascular endothelial cells prevents the leakage of LDL into the retina. In support of this notion, we previously showed that MD2 mediates free fatty acid-induced endothelial cell injury via AMPK/nuclear factor erythroid 2-related factor 2 (Nrf2)^49^. MD2 inhibition in endothelial cells markedly increases the activation of AMPK and the protein levels of Nrf2, suppressing ROS generation^49^. Other researchers have also shown that inhibiting TLR4 attenuates leukocyte accumulation and retinal vascular permeability in diabetic rats^19^. Therefore, these studies suggest that the MD2-TLR4 pathway may mediate different deleterious steps in the complex pathogenesis of diabetic retinopathy.

In summary, using MD2-specific small-molecule inhibitors and MD2 siRNA, we demonstrate that MD2 mediates HOG-LDL-induced TLR4-MyD88 and TLR4-NOX4 signaling pathway activation, oxidative stress, inflammatory responses, and Müller cell apoptosis. We also showed that diabetic mice treated with the MD2 inhibitor L2H17 were protected against retinal injuries. Overall, the study provides evidence for a pathogenic role of MD2 in diabetic retinopathy and supports MD2 inhibition as a feasible strategy for the treatment of diabetic retinopathy.

## Supplementary information

Supplementary file
