# Supplementary material for: MD2 blockade prevents modified LDL-induced retinal injury in diabetes by suppressing NADPH oxidase-4 interaction with Toll-like receptor-4
Source: Exp Mol Med. 2021 Apr 19;53(4):681–94. doi: 10.1038/s12276-021-00607-w (PMC8102522; doi:10.1038/s12276-021-00607-w)
Supplement: Supplementary file 1 — Supplementary file [file 12276_2021_607_MOESM1_ESM.docx]

**Supplementary file**

**MD2 blockade prevents modified LDL-induced retinal injury in diabetes by suppressing NADPH oxidase-4 and its interaction with toll-like receptor-4**

Huaicheng Chen^1,2,#^, Tao Yan^1,#^, Zongming Song^2,3,#^, Shilong Ying^1^, Beibei Wu^1^, Xin Ju^1,2^, Xi Yang^1,2^, Jia Qu^2^, Wencan Wu^2^, Zongduan Zhang^2^, Yi Wang^1,^*

*^1^ Chemical Biology Research Center, School of Pharmaceutical Sciences, Wenzhou Medical University, Wenzhou, Zhejiang, China.*

*^2^ The Eye Hospital of Wenzhou Medical University, Wenzhou, Zhejiang, China.*

*^3^* *Department of Ophthalmology, The Fourth Affiliated Hospital, Zhejiang University School of Medicine, Yiwu, Zhejiang, China.*

*^4^ Henan Eye Institute, Henan Eye Hospital, Henan Provincial People’s Hospital and People’s Hospital of Zhengzhou University.*

^#^ These authors contributed equally to this work.

**Running title:** Activation of NOX4 by modified LDL requires MD2

*** Corresponding author:**

Yi Wang, Ph.D.,

Address: Chemical Biology Research Center, School of Pharmaceutical Sciences, Wenzhou Medical University, Wenzhou 325035, China;

Tel: +86-577-85773060; Fax: +86-577-85773060

E-mail: yi.wang1122@wmu.edu.cn

The supplemental file contains 2 tables and 2 figures.

**Supplementary Table S1.** Primer sequences for real-time qPCR.

| **Gene** | **Species** | **Primer（5’-3’）** |
| --- | --- | --- |
| TNF-α | Human | Forward: CCCAGGGACCTCTCTCTAATC |
|  |  | Reverse: GGGCTACAGGCTTGTCACT |
| IL-1β | Human | Forward: CGCTCCGGGACTCACAGCA |
|  |  | Reverse: TGAGGCCCAAGGCCACAGGT |
| IL-6 | Human | Forward: CACTGGCAGAAAACAACCT |
|  |  | Reverse: TCAAACTCCAAAAGACCAGTGA |
| β-actin | Human | Forward: CTGGCACCCAGCACAAT |
|  |  | Reverse: GCCGATCCACACGGAGTACT |

**Supplementary Table S2.** siRNA sequences for gene konckdown.

| **Gene** | **Species** | **Sequence (5’-3’)** |
| --- | --- | --- |
| MD2 | Human | GAUGCAAGUAUUUCAUACATT |
| NOX4 | Human | ACUGAGGUACAGCUGGAUGUU |
| Control | Human | AUUUCUUUCAUGUUGUGGGTT |

**Supplementary Figure S1. MD2 inhibitors suppress the HOG-LDL-induced H_2_O_2_ levels in MIO-M1 cells.**

MIO-M1 cells were pretreated with L2H17 (5, 10, and 20 μM) or L6H21 (20 μM) for 2 h, followed by the stimulation of HOG-LDL (150 mg/L) for another 3 h. The cells were washed and collected. The levels of H_2_O_2_ in MIO-M1 cells were evaluated using Hydrogen Peroxide Assay kit (Beyotime Biotech, Catalog#: S0038, Nantong China). Quantitative data are presented as mean±SEM, n = 3, ##, *P*<0.01 vs DMSO control group; *, *P*<0.05, **, *P*<0.01; ***, *P*<0.001 vs HOG-LDL treated group.

**
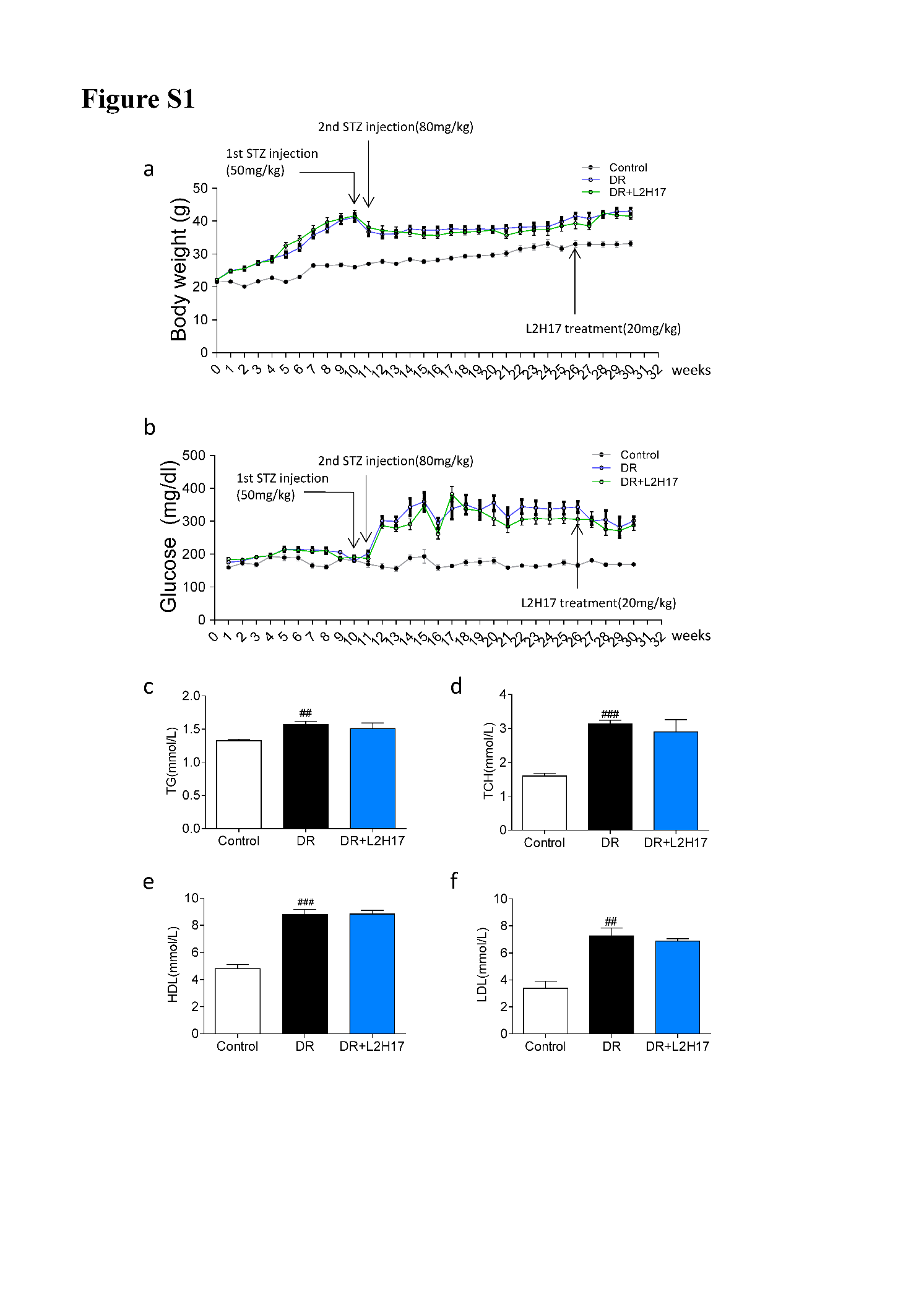
**

**Supplementary Figure S2. Effect of L2H17 on body weights, glucose levels, and serum lipids.**

Control mice were maintained on a high-fat diet for 30 weeks. At weeks 10 and 11, mice were injected with streptozotocin (STZ). Treatment of mice with L2H17 was initiated at week 26. (**a**) Weekly body weight measurements in mice. Timing of STZ administration and L2H17 treatment is indicated. No changes in body weights were noted in diabetic mice treated with L2H17 compared to untreated diabetic mice. (**b**) Blood glucose levels in mice showing no difference upon L2H17 treatment. (**c-f**) Levels of serum triglycerides (TG, **c**), total cholesterol (TCH, **d**), high-density lipoprotein (HDL, **e**), and low-density lipoprotein (LDL, **f**) in mice [Mean ± SEM; n = 4-8; ##*P*<0.01, ###*P*<0.001 compared to non-diabetic control mice].
